# Letter of Concern from the Association of Academic Chairs of Emergency Medicine Regarding ACGME Proposed Changes

**DOI:** 10.5811/westjem.48840

**Published:** 2025-06-27

**Authors:** Richard J. Hamilton, Lance B. Becker, Richard E. Wolfe, D. Adam Algren, Thomas Arnold, Michael Baumann, Ross P. Berkeley, Terrell S. Caffery, Chad M. Cannon, Theodore J. Corbin, Michael E. Chansky, Harinder S. Dhindsa, Charles L. Emerman, David A. Farcy, Chris Fox, Michael A. Gibbs, Christopher S. Goode, Steven “Andy” Godwin, Dietrich Jehle, David Johnson, Samuel M. Keim, Babak Khazaeni, Barry J. Knapp, Clint Hawthorne, John D. Hoyle, Michael Christopher Kurz, Evan Leibner, Robert McNamara, Robert F. McCormack, Edward A. Michelson, Chadwick Miller, Ashley Norse, Andrew Nugent, Brian J. O’Neil, David T. Overton, Edward A. Panacek, William F. Paolo, Denis R. Pauzé, Amanda L. Perez, Ralph J. Riviello, Scott W. Rodi, Peter S. Pang, Juan A. Gonzalez Sanchez, David Seaberg, Adam Schwartz, Stephen A. Shiver, David P. Sklar, Ben C. Smith, Jeffrey R. Stowell, Marc D. Squillante, J. Jeremy Thomas, Terry Vanden Hoek, Gregory A. Volturo, E. Lea Walters, Thomas E. Wyatt, Donald M. Yealy

**Affiliations:** * Crozer Keystone Health System, Department of Emergency Medicine; † Drexel University College of Medicine, Department of Emergency Medicine; ‡ North Shore University Hospital and Long Island Jewish Medical Center, Department of Emergency Medicine; § Donald and Barbara Zucker School of Medicine at Hofstra/Northwell, Institute of Bioelectronic Medicine; || Feinstein Institutes for Medical Research Dorothy and Jack Kupferberg, Department of Emergency Medicine; # Harvard Medical School, Department of Emergency Medicine; ¶ Beth Israel Deaconess Medical Center, Department of Emergency Medicine; ** University Health, Department of Emergency Medicine, Division of Clinical Pharmacology, Toxicology, Therapeutic Innovation; †† Children’s Mercy Kansas City, Department of Pediatric Emergency Medicine; ‡‡ LSU Health Shreveport, Department of Emergency Medicine; §§ Mainhealth, Maine Medical Center, Department of Emergency Medicine; |||| Tufts University School of Medicine, Department of Emergency Medicine; ## University of Nevada, Las Vegas, Kirk Kerkorian School of Medicine at UNLV, Department of Emergency Medicine; ¶¶ LSU, Department of Emergency Medicine; *** University of Kansas Medical Center, University of Kansas Health System, Department of Emergency Medicine; ††† Rush Medical College, Rush University Hospital, Department of Emergency Medicine; ‡‡‡ Cooper Medical School of Rowan University, Cooper University Health Care, Department of Emergency Medicine; ‡‡‡ Virigina Commonwealth, Department of Emergency Medicine; 1 Case Western Reserve, University School Medicine MetroHealth Medical Center, Department of Emergency Medicine; 2 Mount Sinai Medical Center, Department of Emergency Medicine; 3 Herbert Wertheim college of Medicine, Florida International University, Department of Emergency Medicine and Critical Care; 4 University of California, Irvine, Department of Emergency Medicine; 5 Carolinas Medical Center and Levine Children’s Hospital, Department of Emergency Medicine; 6 WVU Medicine, Department of Emergency Medicine; 7 University of Florida College of Medicine - Jacksonville, UF Health Jacksonville, Department of Emergency Medicine; 8 University of Texas Medical Branch, Department of Emergency Medicine; 9 University at Buffalo, Department of Emergency Medicine; 10 Mercy St. Vincents Medical Center, Department of Emergency Medicine; 11 Mel and Enid Zuckerman College of Public Health, Department of Emergency Medicine; 12 University of Arizona College of Medicine - Tucson, Department of Emergency Medicine; 13 Banne r- University Medical Center Tucson, Department of Emergency Medicine; 14 Desert Regional Medical Center, Department of Emergency Medicine; 15 Eastern Virgina Medical School at Old Dominion University, Department of Emergency Medicine; 16 UnityPoint Health Des Moines, Department of Emergency Medicine; 17 Western Michigan University Homer Stryker, MD School of Medicine, Department of Emergency Medicine and Pediatrics and Adolescent Medicine; 18 University of Chicago, Department of Emergency Medicine; 19 Creighton University School of Medicine, Dignity Health Medical Group Chandler, Department of Emergency Medicine; 20 Lewis Katz School of Medicine at Temple University, Temple University Health System, Department of Emergency Medicine; 21 Jacobs School of Medicine and Biomedical Science, University at Buffalo, Department of Emergency Medicine; 22 Buffalo General Medical Center and Erie County Medical Center, Department of Emergency Medicine; 23 Paul L. Foster School of Medicine, Texas Tech University Health Sciences Center El Paso, Department of Emergency Medicine; 24 Wake Forest University School of Medicine, Department of Emergency; 25 University of Arkansas Medical Center, Department of Emergency Medicine; 26 University Of Iowa Health Care, Department of Emergency Medicine; 27 Albany Medical Center, Department of Emergency Medicine; 28 University of South Alabama, Department of Emergency Medicine; 29 Upstate Medical University Syracuse NY, Department of Emergency Medicine; 30 Albany Medical Center, Department of Emergency Medicine; 31 The Permanente Medical Group, Department of Emergency Medicine; 32 Manteca/Modesto Central Valley Service Area, Department of Emergency Medicine; 33 Joe R. and Teresa Lozano Long School of Medicine, UT Health San Antonio University Health System, Department of Emergency Medicine; 34 Geisel school of Medicine at Dartmouth, Dartmouth Hitchcock Medical Center, Department of Emergency Medicine; 35 Indiana University School of Medicine, Department of Emergency Medicine; 36 UPR School of Medicine, Department of Emergency Medicine; 37 US Acute Care Solutions, Academic Division; 38 Southern California Permanente Medical Group, Department of Emergency Medicine; 39 Kaiser Permanente San Diego, Department of Emergency Medicine; 40 Medical University of GA at Augusta University, Department of Emergency Medicine; 41 University of New Mexico, Department of Emergency Medicine; 42 ASU Health, College of Health Solutions, Division of Medicine; 43 University of Tennessee College of Medicine, Department of Emergency Medicine; 44 The University of Arizona, College of Medicine - Phoenix, Department of Emergency Medicine; 45 University of Illinois College of Medicine Peoria, Department of Emergency Medicine; 46 University of Louisville School of Medicine, University of Louisville Health, Department of Emergency Medicine; 47 University of Illinois College of Medicine, University of Illinois Health, Department of Emergency Medicine; 48 University of Illinois Chicago, Center for Advanced Resuscitation Medicine Center for Cardiovascular Research; 49 UMass Chan Medical School, UMass Memorial Health, Department of Emergency Medicine; 50 Loma Linda University Health, Department of Emergency Medicine; 51 U of Minnesota Medical School, Department of Emergency Medicine; 52 Hennepin County Medical Center, Department of Emergency Medicine; 53 University of Pittsburgh School of Medicine, Department of Emergency Medicine

## Abstract

This letter, signed by over 50 academic chairs of emergency medicine, urges the ACGME to reconsider a proposed mandate requiring all emergency medicine residency programs to adopt a four-year training model. The authors argue that current three-year programs are supported by data demonstrating equivalent educational and clinical outcomes compared to four-year formats. They criticize the flawed survey methodology underpinning the proposal, note the loss of milestone-based training flexibility, and highlight the lack of added scholarly or clinical value in the fourth year. The letter also outlines negative consequences for fellowship participation, workforce development, trainee debt, and diversity. The signatories advocate for maintaining the current flexible training model to preserve excellence, equity, and innovation in emergency medicine education.

To: Accreditation Council for Graduate Medical Education

401 North Michigan Avenue, Suite 2000

Chicago, IL 60611

May 1, 2025

Dear Members of the Accreditation Council for Graduate Medical Education,

We, the undersigned academic chairs of emergency medicine departments are writing to express our deep concerns regarding the recent proposal to mandate a transition from the current 3- or 4-year training format to a compulsory 4-year residency requirement in Emergency Medicine. This decision would impact 80% of all Emergency Medicine training programs and is of utmost concern to us.

Our objections to this decision are as follows:

Data from the American Board of Emergency Medicine and other reputable sources does not support the necessity of lengthening training to four years to achieve the knowledge thresholds sought. In fact, existing data supports the quality and competency of residents graduating from well-established 3-year programs. Mandating an additional year of training without clear empirical evidence undermines the proven efficacy of current training models. For example, studies by Beeson et al.1, Hayden et al.2, and Nikolla et al.3 demonstrate that there are no significant differences in board examination performance or clinical competence between graduates of 3-year and 4-year programs.

The decision to implement this change appears to have been based on survey data that is fundamentally flawed. The survey methodology obscured the true intent behind the questions and included leading questions that biased the results. Such flawed data cannot serve as a reliable basis for making substantial changes to residency training requirements.

By mandating a 4-year training period, the new policy negates the milestone-based curriculum that has been a cornerstone of Emergency Medicine residency training. Program Directors currently have the discretion to graduate residents after 3 years upon achievement of all required milestones or to add individualized training as needed. This flexibility allows for tailored educational experiences that align with individual resident capabilities and career goals. A one-size-fits-all approach would erode this effective, individualized training model.

Moreover, the addition of a fourth year fails to introduce meaningful enhancements to scholarly activity or research requirements that would substantively advance our specialty. While we recognize that extended training may benefit select individuals, mandating a 4-year format for all residents should be accompanied by corresponding scholarly expectations and justifications—not just additional service time.

While Emergency Medicine physicians are well-prepared for careers beyond direct clinical emergency department care through the current 3-year format, mandating a fourth year is less efficient for learners, the specialty, and the healthcare workforce. Residents seeking non-clinical pathways typically pursue fellowships, and a streamlined 3-year residency followed by focused fellowship training offers the most efficient trajectory. A study by Ehmann et al.4 revealed that transitioning from a 3-year to a 4-year Emergency Medicine residency format led to a reduction in the number of graduates pursuing fellowship training. This suggests that extended base training may dissuade residents from subspecializing, potentially diminishing the future pipeline of EM experts in critical areas such as toxicology, ultrasound, and critical care. Imposing a fourth generalized year risks discouraging fellowship participation and contradicts ongoing efforts to reduce unnecessary burdens on trainees. Maintaining the 3-year model supports individualized training, fosters lifelong learning and career satisfaction, and sustains a diverse and dynamic pipeline for future Emergency Medicine leadership.

The financial impact of transitioning to a mandatory 4-year model is also significant. For hospitals, adding an incompletely funded fourth year would further strain already thin margins threatened by reimbursement cuts. Arguments suggesting that increased low-acuity clinical time and reduced advanced practitioner staffing would offset these costs conflate service needs with educational priorities, undermining the true mission of graduate medical education. For trainees, the additional year would exacerbate their already considerable six-figure educational debt—a burden that disproportionately affects URiM (Underrepresented in Medicine) students and risks dissuading them from entering the specialty altogether.

In conclusion, we urge the ACGME to reconsider this decision in light of these concerns. Flexibility in residency training duration, grounded in evidence-based practices and responsive to the diverse needs of our trainees and specialty, is essential to advancing Emergency Medicine education and maintaining excellence in patient care.

Thank you for considering our perspectives on this critical matter. We remain committed to working collaboratively with the ACGME to ensure that any changes to Emergency Medicine residency requirements uphold the highest standards of medical education and service to our communities.

Sincerely,
